# Complete Genomic Sequences of H3N8 Equine Influenza Virus Strains Used as Vaccine Strains in Japan

**DOI:** 10.1128/genomeA.00172-18

**Published:** 2018-03-22

**Authors:** Manabu Nemoto, Takashi Yamanaka, Hiroshi Bannai, Koji Tsujimura, Hiroshi Kokado

**Affiliations:** a Equine Research Institute, Japan Racing Association, Tochigi, Japan

## Abstract

We sequenced the eight segments of influenza A virus strains A/equine/Ibaraki/1/2007 and A/equine/Yokohama/aq13/2010, which are strains of the Florida sublineage clades 1 and 2 of the H3N8 subtype equine influenza virus. These strains have been used as vaccine strains in Japan since 2016 in accordance with World Organization for Animal Health (OIE) recommendations.

## GENOME ANNOUNCEMENT

Equine influenza virus (EIV) (family Orthomyxoviridae, genus Influenzavirus A) causes the acute respiratory disease referred to as equine influenza (EI) (1). Two subtypes of influenza virus, H7N7 and H3N8, have been isolated from horses; however, the H7N7 subtype is considered to be extinct (2). In contrast, the H3N8 subtype has caused many outbreaks of EI among horses throughout the world (3). The H3N8 subtype of EIV has diverged into several lineages, and two clades of the Florida sublineage, clades 1 (Fc1) and 2 (Fc2), have prevailed in recent years (1, 4, 5). Fc1 viruses are predominant in the United States, while Fc2 viruses are predominant in Europe. Therefore, since 2010, the World Organization for Animal Health (OIE) has recommended that EI vaccines include both clades (6). In accordance with OIE recommendations, A/equine/Ibaraki/1/2007 (Fc1) and A/equine/Yokohama/aq13/2010 (Fc2) have been used in Japan as vaccine strains since 2016 (7). The hemagglutinin (HA) and neuraminidase (NA) sequences of the two strains have been released, but the genome sequences of the other six segments have not been reported. Here, we report the complete genome sequences of Japanese vaccine strains A/equine/Ibaraki/1/2007 (8) and A/equine/Yokohama/aq13/2010 (9).

Strains A/equine/Ibaraki/1/2007 and A/equine/Yokohama/aq13/2010 were passaged six and four times, respectively, in 10-day-old embryonated hens’ eggs. Viral RNA was extracted using a MagNA Pure LC total nucleic acid isolation kit (Roche Diagnostics, Mannheim, Germany). The cDNA was synthesized using the Uni12 primer (5′-AGCAAAAGCAGG-3′) and Superscript III reverse transcriptase (Thermo Fisher Scientific, Waltham, MA, USA). Amplification of the eight gene segments was performed by using PCR with KOD-Plus-Neo DNA polymerase (Toyobo, Osaka, Japan) and the universal primer sets described by Hoffmann et al. (10). The amplified products of the two strains were sequenced using next-generation sequencing technology on the Ion PGM system (Thermo Fisher Scientific) according to the manufacturer’s instructions. The raw signal data were analyzed by using Torrent Suite version 5.6.0 based on known EIV sequences.

The sequences of the three polymerase proteins (PB2, PB1, and PA), nucleoprotein (NP), NA, matrix (M), and nonstructural protein (NS) of both strains comprised 2,308, 2,309, 2,200, 1,529, 1,424, 991, and 855 nucleotides, respectively, excluding the primer sequences. The HA sequences of A/equine/Ibaraki/1/2007 and A/equine/Yokohama/aq13/2010 comprised 1,728 and 1,734 nucleotides, respectively. BLASTn analysis showed that all eight segments of A/equine/Ibaraki/1/2007 had more than 99% nucleotide identities to those of A/equine/Tottori/1/07 (Fc1) (GenBank accession numbers AB591842 to AB591849) (11). In addition, the seven segments other than the NS of A/equine/Ibaraki/1/2007 were closely related with those of A/equine/Kyonggi/SA1/2011 (Fc1) (GenBank accession numbers JX844143 to JX844149). Strain A/equine/Kyonggi/SA1/2011 had a deletion in NS (12). All eight segments of A/equine/Yokohama/aq13/2010 had more than 99% nucleotide identities with those of A/equine/Richmond/1/2007 (GenBank accession numbers FJ195395, FJ195429, and KF559332 to KF559337), which has been a Fc2 vaccine strain recommended by OIE (6).

In this study, we revealed the complete genomic sequences of A/equine/Ibaraki/1/2007 (Fc1) and A/equine/Yokohama/aq13/2010 (Fc2), which are EI vaccine strains in Japan. The results of this study will be useful for evaluating current vaccines and for selecting future ones.

### Accession number(s).

The complete genome sequences of A/equine/Ibaraki/1/2007 (H3N8) and A/equine/Yokohama/aq13/2010 (H3N8) have been deposited in GenBank/EMBL/DDBJ under accession numbers LC369069 to LC369084.
